# Investigating psychosocial factors and systemic inflammation using dried blood spots: a scoping review

**DOI:** 10.1007/s00127-025-02941-0

**Published:** 2025-06-13

**Authors:** William A. H. Schwartz, Tyler A. Jacobson, Ramzy P. Issa, Q. Eileen Wafford, Denise A. Nunes, William E. Funk, Gregory E. Miller, Thomas W. McDade, Joseph M. Feinglass

**Affiliations:** 1 Department of Psychiatry, Lenox Hill Hospital, New York, NY 10075 USA; 2 Department of Preventive Medicine, Feinberg School of Medicine, Northwestern University, 420 E. Superior Street, Chicago, IL 60611 USA; 3 Department of Medicine, Medical College of Wisconsin, Milwaukee, WI 53226 USA; 4 Galter Health Sciences Library, Northwestern University Feinberg School of Medicine, Chicago, IL 60611 USA; 5 Department of Psychology, Weinberg College of Arts and Sciences, Northwestern University, Evanston, IL 60611 USA; 6 Institute for Policy Research, Northwestern University, Evanston, IL 60208 USA; 7 Department of Anthropology, Northwestern University, Evanston, IL 60208 USA; 8 Division of General Internal Medicine, Northwestern University Feinberg School of Medicine, Chicago, IL 60611 USA

**Keywords:** Dried blood spots, Psychosocial factors, Stress, Inflammation

## Abstract

**Purpose:**

This scoping review has two primary aims: (1) to synthesize U.S.-based studies utilizing dried blood spot (DBS) sampling to examine associations between psychosocial factors and systemic inflammation, as measured by C-reactive protein (CRP), and (2) to evaluate the methodological utility of DBS for advancing inclusive, population-based biomarker research. We explore DBS adoption, variability in methods and findings, and how this approach can enhance understanding of health disparities driven by psychosocial stressors.

**Methods:**

A comprehensive search of databases including MEDLINE, Embase, Cochrane Library, CINAHL Plus, and PsycINFO was conducted. Inclusion criteria encompassed U.S.-based studies employing DBS sampling to quantify CRP in relation to psychosocial factors. Studies were categorized into four domains: socioeconomic and neighborhood contexts, adverse life events and trauma, social experiences and interpersonal dynamics, and mental health and emotional well-being. Data were extracted, synthesized, and narratively analyzed.

**Results:**

Thirty studies met inclusion criteria, with sample sizes ranging from 20 to 13,236. Key findings indicated strong associations between psychosocial stressors (e.g., discrimination, social isolation) and elevated CRP. Few studies conducted sex- or gender-based analyses. DBS was particularly effective for research in underrepresented populations, enabling cost-effective, minimally invasive sampling in diverse and resource-limited settings.

**Conclusion:**

This review highlights DBS as an important tool for psychiatric biomarker research, offering scalability and inclusivity. Findings affirm the link between psychosocial factors and systemic inflammation, suggesting avenues for targeted interventions. Future research should explore additional biomarkers and psychosocial determinants with sex stratified analyses to better understand inflammation-related health disparities.

**Supplementary Information:**

The online version contains supplementary material available at 10.1007/s00127-025-02941-0.

## Introduction

Psychosocial stressors have a well-documented impact on physical health, particularly through inflammatory pathways implicated in chronic diseases such as cardiovascular disease (CVD). Stress biology research shows that chronic psychosocial stress—arising from factors like socioeconomic disadvantage and discrimination—can disrupt homeostasis, leading to dysregulation in immune function and systemic inflammation. A key marker in this pathway is C-reactive protein (CRP), a liver-produced inflammatory biomarker triggered by interleukin-6 (IL-6) and other cytokines, and linked to elevated risk of CVD, metabolic syndrome, and related conditions [1, 2]. Notably, in the United States pronounced socioeconomic status (SES) disparities in CRP levels have been reported, highlighting the role of systemic inflammation in mediating the health effects of social determinants (Alley et al. 2006, [3].

### The stress biology model and its relevance to inflammation

The stress biology model offers a framework for understanding how chronic psychosocial stress influences health through physiological pathways. Models such as the Allostatic Load Model [4], the Biopsychosocial Model [5], and the Biological Embedding of Childhood Adversity Model [6] explain how stressors like social isolation, low SES, and discrimination activate the hypothalamic-pituitary-adrenal (HPA) axis and sympathetic nervous system. This leads to cortisol secretion, immune cell mobilization, and heightened immune responsivity, potentially resulting in sustained low-grade inflammation [6]. Notably, these pathways may differ by sex and gender, with studies highlighting variations in HPA axis reactivity, cytokine signaling and inflammatory biomarker expression, likely due to both hormonal regulation and sociocultural context [7]

Inflammation from these pathways is often measured by CRP, a sensitive and widely used biomarker of systemic inflammation and predictor of CVD risk [8, 9]. Early CRP assays had limited sensitivity, with thresholds such as 3 mg/L or 8 mg/L shaped by assay constraints. These categorical cutoffs used to stratify individuals into CVD risk groups originated from the technical constraints of early measurement methods [10]. With the advent of high-sensitivity CRP (hs-CRP) assays, it became possible to detect CRP at concentrations as low as 0.1 mg/L, enabling analysis as a continuous variable—preferred in epidemiologic research for preserving variability and detecting subtle associations [11–14]. Nonetheless, categorical risk stratification cutoffs—<1 mg/L (low risk), 1–3 mg/L (moderate risk), and >3 mg/L (high risk)—remain widely used for clinical interpretation and research [15], helping to contextualize chronic inflammation in disease risk.

Extensive evidence links psychosocial stressors to elevated CRP. For example, individuals experiencing chronic social isolation and low social support exhibit higher CRP levels, suggesting that social connectedness is a significant modulator of inflammation and immune function [16, 17]. Discrimination and racism are similarly linked to elevated CRP, reflecting the immunological toll of chronic vigilance [18, 19]. These findings align with life course models emphasizing the biological embedding of adversity over time [20], [21].

### The life course health development framework

The Life Course Health Development (LCHD) model explains how psychosocial stressors influence health trajectories over time [22], Kuh & Ben-Shlomo [23]. It posits that early-life exposures to stress, disadvantage, and adverse social conditions can shape biological development in ways that increase the risk of chronic disease in adulthood [21]. Health, therefore, is viewed as a dynamic process molded by cumulative experiences across the lifespan. Timing is critical—stress during sensitive developmental periods may have lasting effects on immune function and inflammatory responses (Shonkoff et al. 2012). Accordingly, the model suggests that addressing early-life stressors could substantially reduce long-term CVD risk and inflammatory burden [20], [24], [25].

Research within this framework identifies low SES as a key driver of elevated CRP. Individuals in disadvantaged environments are more likely to encounter stressors that activate inflammatory pathways. SES-based gradients in CRP persist across the life course, with childhood SES strongly predicting adult CRP levels—even after adjusting for adult SES [26], Pollitt et al. 2008). This cumulative pattern underscores the role of structural inequities in shaping inflammation-related health disparities [27].

### Dried blood spot sampling: a valuable tool for inflammatory research

Dried blood spot (DBS) sampling is an innovative and cost-effective approach to measuring inflammatory biomarkers, particularly in large-scale epidemiologic studies. Collected via finger prick onto filter paper, DBS samples can be dried, stored, and transported at ambient temperatures—facilitating research in diverse and hard-to-reach populations. Compared to venipuncture, DBS does not require trained phlebotomists and is less expensive to process and store [28, 29]. It also enables at-home self-collection, with samples mailed to laboratories [29], supporting scalable and accessible study designs [30].

DBS is particularly useful for studying biomarkers in underserved populations, where traditional venous blood draws pose logistical barriers and may limit participation. Its portability and acceptability support broader demographic reach, improving inclusiveness and generalizability [31]. DBS samples can be stored long-term at room temperature without compromising integrity, making the method especially suited for remote and resource-limited settings [28].

While this review emphasizes CRP, DBS assays have been developed for other inflammatory biomarkers, including IL-6, tumor necrosis factor-alpha (TNF-α), and serum amyloid A [32]. Several of these show acceptable correlation with serum-based assays and can be measured using multiplex platforms adapted for DBS [33]. Although CRP remains the most validated, the expanding ability to quantify multiple markers from a single DBS sample highlights the growing utility of this method in immuno-epidemiological research.

### Current gaps and the purpose of the present study

Although numerous studies have examined links between psychosocial factors and CRP, no comprehensive synthesis exists of U.S.-based research using DBS sampling. CRP was selected due to its reliability as a marker of systemic inflammation, strong associations with cardiovascular and metabolic disease, and widespread use in both clinical and research contexts [34]. Focusing on a single inflammatory biomarker enables a coherent synthesis of study designs and findings. The U.S. context—marked by stark health disparities, including life expectancy gaps exceeding 20 years across demographic groups [35]—was chosen for its unique social, economic, and healthcare dynamics. These structural inequities shape the relationship between psychosocial stressors and inflammation. Unlike low- and middle-income countries (LMICs), where infections and malnutrition may confound CRP levels, the U.S. provides a clearer setting for examining these associations [36].

We therefore conducted a scoping review to systematically map the landscape of epidemiological studies using DBS to explore the relationship between psychosocial stressors and inflammation. This review has two primary goals:

1. To synthesize how psychosocial stressors are associated with CRP in U.S.-based studies utilizing DBS methodology
2. To evaluate the methodological utility of DBS for biomarker collection among underrepresented and hard-to-reach populations.

These aims highlight the scientific contributions of DBS-enabled research and its potential to expand equity and inclusion in psychosocial biomarker studies.

## Methods

A search strategy was collaboratively developed by medical librarians (QEW, DAN) and review authors (WAS, TAJ), incorporating keywords and controlled vocabulary to identify studies related to DBS sampling, inflammatory biomarkers, CVD, and psychosocial factors. Searches were conducted in MEDLINE (Ovid), Embase (Elsevier), the Cochrane Library (Wiley), CINAHL Plus with Full Text (EBSCO), and PsycINFO (EBSCO) on February 25, 2022, and updated on April 24, 2024. Full search details are available in the **Supplementary Information**.

Titles and abstracts were screened in duplicate using Rayyan by two review authors (WAS, TAJ). Inclusion criteria required studies to: (1) use DBS to quantify CRP, (2) measure a psychosocial factor, (3) be population-based (not methods or assay validation), and (4) be conducted in the United States. Studies published before 2000 were excluded due to outdated analytical methods.

Included studies were categorized by primary psychosocial domain: socioeconomic and neighborhood contexts; adverse life events and trauma; social experiences and interpersonal dynamics; or mental health and emotional well-being. Studies spanning multiple domains were assigned based on their primary research focus to ensure consistency in synthesis.

Key study characteristics were extracted by review authors (WAS, TAJ, RI), with all data spot-checked by a third reviewer. These details are summarized in Table 1. A narrative synthesis was conducted to describe study designs and highlight significant findings.

**Table 1:** Summary of US-based studies using DBS sampling for CRP biomarker measurements in relation to psychosocial factors

Author (Year)	Psychosocial factor(s)	N	Study population (location)	Study design	Years of follow up	Sample collection method	Primary outcomes	Sex/Gender Analysis
*Socioeconomic and neighborhood contexts*								
Boch et al. [37]	Parental incarceration	12,843 (11,843 for maternal incarceration; 10,816 for paternal incarceration)	Young adults, aged 24–32 years at wave 4 (Nationally representative sample)	Longitudinal cohort study (National Longitudinal Study of Adolescent to Adult Health; Add Health)	14 years (wave 1: 1994-1995, wave 4: 2007-2008)	Field interviewers during in-home interviews	Exposure to paternal incarceration during childhood was associated with increased odds of low-grade inflammation (CRP levels of 3–10 mg/L) among adult women This relationship was stronger for women who experienced paternal incarceration before the age of 18 or who experienced frequent paternal incarceration	Sex-stratified models showed significant associations between paternal incarceration and elevated CRP in females but not males Maternal incarceration was not associated with CRP in either group. Models controlled for pregnancy and hormonal contraceptive use in women
Chiang et al. [38]	Parental education, household income *Secondary factors:* Positive affect, Negative affect, Negative interactions	316	Adolescents, ages 14–20 (Los Angeles, CA)	Cross-sectional study	N/A	Obtained by researchers during initial home or research center visits	Lower parental education was associated with higher CRP levels Positive affect, but not negative affect or social interactions, mediated this association. Adolescents with lower SES exhibited lower daily positive affect, which predicted higher CRP levels	Sex was included as a covariate in all models, but no sex-stratified analyses were reported The study did not assess whether the associations between SES, affect, and CRP differed by sex
Curci et al. [39]	Neighborhood disadvantage	322 mother-child dyads 181 completed 7.5-year laboratory follow-up	Low-income Mexican American mother-child dyads, mothers aged 18–42 years at baseline (Southwestern United States)	Longitudinal cohort study	7.5 years	Study staff during laboratory visits	Higher prenatal neighborhood disadvantage indirectly predicted higher CRP in children at age 7.5 years	Biological sex (child sex) was collected and tested as a covariate but was not significantly associated with any outcome variables Final models did not include sex to preserve parsimony. No interaction or stratified analyses by sex or gender were conducted
Freeman et al. [40]	Perceived (subjective) Social Status	13,236	Young adults, 24–32 years (Nationally representative sample)	Longitudinal cohort study (National Longitudinal Study of Adolescent to Adult Health; Add Health)	14 years (wave 1: 1994-1995, wave 4: 2007-2008)	Field interviewers during in-home interviews	Lower subjective social status (SSS) was associated with higher CRP in young adults	Performed detailed sex-stratified analyses. Found that SSS was significantly associated with CRP in men but not in women after adjusting for objective SES and other covariates A significant SSS × sex interaction persisted across sensitivity analyses, including exclusion of pregnant participants and those using hormonal contraceptives
Holmes et al. [41]	Neighborhood disorder, Neighborhood social capital	151 (subset with CRP measurement)	Foreign born Brazilian adults (Boston metropolitan area)	Cross-sectional study (Boston Metropolitan Immigrant Health & Legal Status Survey)	N/A	Field interviewers during in-home interviews	Higher neighborhood disorder was associated with elevated CRP levels, while greater neighborhood social capital was associated with reduced CRP levels Unauthorized migrants were more likely to have elevated CRP than their legal counterparts	Sex was included as a covariate in multivariate models; women were significantly more likely to have high CRP than men, with a 31% lower likelihood observed among males No further gender-based stratification or interaction analyses were reported
McDade et al. [42]	Educational attainment, Household wealth *Secondary factors:* Depressive symptoms, anxiety, perceived stress	1,580	Middle-aged and older adults, ages 57–85 (Nationally representative sample)	Cross-sectional study (National Social Life, Health, and Aging Project)	N/A	Field interviewers during in-home interviews	Higher educational attainment (post-college schooling) predicted lower CRP Higher household wealth (> $500,000+) predicted lower CRP	CRP levels were higher in females in unadjusted models but not significant after adjusting for SES and health factors No sex-stratified models were reported
Nguyen et al. [43]	Neighborhood physical advantage, Neighborhood social cohesion *Secondary factors:* Hopelessness, Pessimism	1,004	Older non-Hispanic Black adults, age ≥ 60 (Nationally representative sample)	Cross-sectional study (part of Health and Retirement Study)	N/A	Field interviewers (unspecified location)	No association was found between neighborhood physical disadvantage or social cohesion and CRP However, an association between neighborhood physical disadvantage and CRP was moderated by hopelessness and pessimism	Gender (binary) included as a covariate in regression models, but no sex- or gender-stratified analyses reported and no interactions tested by sex/gender
Schmeer et al. [44]	Home physical environment	353	Children, ages 3–18 (Los Angeles metro area, CA)	Cross-sectional study (Los Angeles Family and Neighborhood Survey)	N/A	Phlebotomists	Children living in homes with more low-quality physical environmental indicators (e.g., clutter, crowding, uncleanliness) had significantly higher levels of CRP with the association being strongest for younger children Each additional low-quality home environment indicator was associated with an 11% increase in CRP	Sex was included as a covariate in all models, and boys were found to have significantly lower CRP levels than girls However, no sex-specific analyses or interaction terms were conducted to explore differential associations between home environment and CRP by sex
*Adverse life events and trauma*								
John-Henderson et al. [45, 46]	Childhood adversity	46	Young adults, ages 18–30 (Northwestern United States)	Experimental study with participants randomly assigned to either a sleep restriction group or a control group	N/A	Participant self-collection	Childhood adversity amplified the inflammatory response to sleep restriction with participants with high levels of childhood adversity showing significant increases in CRP levels after sleep restriction, whereas those with low levels of childhood adversity not exhibiting this response	Over 80% of the sample identified as female; however, no sex-stratified analyses were conducted and sex was not included as a moderator or covariate in analyses of CRP outcomes
Méndez Leal et al. [47]	Early life stress	89 (subset with CRP measurement)	Pregnant women in or near their third trimester, ages 19–44 years (three sites: Washington, DC; Lake County, IL; and eastern North Carolina)	Cross-sectional study (Community Child Health Network longitudinal study)	N/A	Trained research staff during home visits	Early life stress was not found to be significantly associated with elevated CRP in pregnant women in this study	N/A; sample consisted entirely of pregnant women However, the study examined inflammation in the context of gendered physiological and social experiences, including early life stress and pregnancy. Authors note that 78% of participants had CRP levels >3 mg/L but found no direct association between early life stress and CRP in this female sample
Yuan et al. [48]	Early life stress	83	Adolescents, ages 13–17 years (Bay area, US)	Cross-sectional study	N/A	Study staff during laboratory visits	No direct association was seen between severity of early life stress (ELS) and CRP; however, ELS moderated associations of CRP with neural activation during implicit emotion regulation	CRP analyses included sex as a covariate but did not report stratified or interaction results by sex Authors noted sex differences in CRP were not statistically significant No analyses were conducted to assess gender identity or gendered experiences
*Social experiences and interpersonal dynamics*								
Cook et al. [49]	Daily discrimination	20	Young sexual and gender minority men (YSMM), ages 18 to 35 years (New York Tri-State area)	Cross-sectional study	N/A	Participant self-collection (HemaSpot device)	No statistically significant association was found between daily discrimination and CRP in YSMM	N/A; study included only cisgender young sexual minority men
Copeland et al. [50]	Bully-victim experiences	1,420	Children/adolescents, ages 9–16 at intake (Western North Carolina)	Longitudinal, cohort study (Great Smoky Mountains Study)	Up to 12 years (participants were followed annually until age 16, with additional assessments at ages 19 and 21)	Obtained by researchers during in-person assessments	Children who were victims of bullying had higher CRP levels as adults, with a dose-response relationship based on the cumulative number of bullying exposures. Conversely, pure bullies who were never victims exhibited lower CRP levels compared to uninvolved peers, suggesting a protective effect linked to higher social status from bullying behavior	Sex was included as a covariate in all regression models predicting CRP Stratified results by sex were not reported, but the study controlled for sex in assessing associations between bullying roles (victim, bully, bully–victim) and CRP trajectories from childhood to adulthood
Cudjoe et al. [51]	Social isolation	4,648	Medicare beneficiaries, ages ≥ 65 years (Nationally representative sample)	Cross-sectional study (National Health and Aging Trends Study)	N/A	Obtained by researchers during in-person assessments	Social isolation was significantly associated with elevated levels of CRP A dose-response relationship was observed, with severe social isolation correlating with higher CRP levels	Analyses adjusted for gender as a covariate, and sample characteristics were disaggregated by gender However, no sex-stratified results were reported, and gender was not examined as an effect modifier in the relationship between social isolation and CRP
Drolet et al. [52]	Perceived racism	118 (subset with CRP measurement)	African American adults, ages 18–69 years (Metro Detroit, MI)	Cross-sectional study	N/A	Study staff during laboratory visits	No significant associations or interactive effects between CRP and perceived racism, racial centrality, or religious intensity However, the positive association between perceived racism and CRP, as well as the positive association between racial identity and CRP, approached significance	CRP analyses adjusted for sex; no main or interaction effects by sex were reported Authors noted sex was a significant covariate in predicting CRP but did not explore sex-specific associations
Goosby et al. [53]	Perceived discrimination	58	Low-income African American youth, ages 10–15 (Omaha, NE)	Cross-sectional study (Omaha Urban Research on Health Study; OURHealth Study)	N/A	Obtained by researchers during in-person assessments	Perceived discrimination among low-income African American youth was positively associated with elevated levels of CRP	Sex (female) was included as a covariate in regression models, but no sex-stratified results were reported Approximately 79% of the youth sample was female, and the authors note the need for more sex-balanced studies to enhance generalizability
Guardino et al. [54]	Financial stress, Chronic life stress, Everyday racial discrimination, Interpersonal violence, Perceived stress	1,206	Postpartum women, ages 18–40 years (5 sites: Washington, DC (urban); Baltimore, MD (urban); Los Angeles, CA (urban); Lake County, IL (suburban); eastern North Carolina (rural))	Longitudinal study (Community Child Health Network study; CCHN)	1 year	Field interviewers during in-home interviews	Chronic financial stress at 1-month postpartum predicted higher levels of CRP at 6 and 12 months postpartum	N/A; All study participants were postpartum women
Lee et al. [55]	Social media use (SMU)	115 enrolled and completed phase 1 66 completed phase 2 laboratory follow-up after 5 weeks	Undergraduate students, age 18–44 (Midwest, US)	Short-term longitudinal study	5 weeks	Study staff during laboratory visits	Objective SMU was positively associated with CRP both cross-sectionally and longitudinally Average weekly SMU between phases 1 and 2 predicted increased CRP levels, demonstrating a cumulative effect of social media use on inflammation over time	Sex was included as a covariate in all regression models, but no stratified or interaction analyses were conducted to examine differential associations by sex or gender
McQuillan et al. [56]	Gender-based stressors, Gender-based supports	50 (subset with CRP measurement)	Transgender and gender-nonconforming youth (TGNC) youth, ages 9–20 years (Midwest, US)	Cross-sectional study	N/A	Obtained by researchers during clinic visits	Higher composite Gender Minority Stress and Resilience (GMSR) score(indicating higher gender-based stressors and lower gender-based support) predicted higher CRP No significant relationships were found between individual subscales of gender minority stress or support and CRP levels	Analyses included comparisons by gender identity (transfeminine vs. transmasculine); CRP was not significantly different by gender Two nonbinary participants were excluded from final analysis due to limited sample size
*Mental health and emotional well-being*								
Blevins et al. [57]	Positive affect, Perceived psychological stress	3,093	Young adults, ages 25–34 years (Nationally representative sample)	Cross-sectional study (data from wave IV of the National Longitudinal Study of Adolescent to Adult Health; Add Health)	N/A	Field interviewers during in-home interviews	Perceived psychological stress (PPS) and positive affect (PA) as individual predictors did not have significant association with CRP; however, PPS and PA significantly interacted to predict levels of CRP, indicating PA was protective against elevated CRP in those with elevated PPS	Analyses adjusted for sex, and sex was significantly associated with CRP in bivariate models (higher CRP in females) However, no sex-stratified or interaction analyses were reported
Chiang et al. [58]	Perceived stress, depressive symptoms, major life events, daily interpersonal stress	350 at baseline, with follow-up data from 248 participants at Wave 2 and 180 participants at Wave 3	Adolescents, ages 14–20 (Los Angeles, CA)	Longitudinal study (three waves of data collection over 4 years)	4 years, with assessments every 2 years	Field interviewers during in-home interviews	CRP levels increased steadily from mid-adolescence into young adulthood Within-person fluctuations in perceived stress were positively associated with higher CRP levels, whereas average levels of stress, major life events, and daily interpersonal stress did not significantly influence CRP trajectories	Included exploratory analyses of gender differences in CRP trajectories and stress-CRP associations No significant gender differences were found in CRP levels, their age-related change, or in within- or between-person associations with stress
Costello et al. [59]	Drug abuse/dependence	1,420	Children/adolescents, ages 9–16 at intake (Western North Carolina)	Longitudinal, cohort study (data from the Great Smoky Mountains Study; GSMS)	Up to 12 years (participants were followed annually until age 16, with additional assessments at ages 19 and 21)	Obtained by researchers during in-person assessments	Nicotine use in childhood/adolescence predicted higher CRP in young adulthood	Sex included as a covariate in multivariate models predicting CRP CRP levels were significantly higher in females, but sex differences were not analyzed as a primary exposure or moderator
Copeland et al. [60]	Depressive symptoms, Cumulative depressive episodes	1,420	Children/adolescents, ages 10–16 (Western North Carolina)	Longitudinal, cohort study (data from the Great Smoky Mountains Study; GSMS)	Up to 12 years (participants were followed annually until age 16, with additional assessments at ages 19 and 21)	Obtained by researchers during in-person assessments	Cumulative depressive episodes during childhood and adolescence significantly predicted higher CRP levels in young adulthood	Analyses adjusted for sex and tested associations between depression and CRP across the full sample; descriptive statistics showed higher rates of depressive symptoms and diagnoses among females, but sex-stratified analyses were not reported
Guimond et al. [61]	Sense of purpose in life	6,925	Older adults, ages > 50 years (Nationally representative sample)	Longitudinal study (part of Health and Retirement Study)	8 years, with data collected at baseline and two follow-up waves (baseline: 2006/2008, follow-ups: 2010/2012 and 2014/2016)	Field interviewers during in-home interviews	Higher versus lower sense of purpose is associated with lower hazards of developing elevated CRP in older men; no significant association found in older women	Sex-stratified models tested; higher purpose was associated with reduced CRP risk in men but not women Interaction between purpose and sex was not statistically significant (p = 0.15), but associations were stronger and more consistent in men across models
Ironson et al. [62]	Positive affect, life satisfaction	1,979	Adults, ages 18–96 years (Nationally representative sample)	Cross-sectional study (data from Landmark Spirituality and Health Survey; LSHS)	N/A	Obtained by researchers during in-person assessments	Higher positive affect and life satisfaction were significantly associated with lower CRP levels Individuals with low positive affect or low life satisfaction had higher odds of elevated CRP (≥3 mg/L)	Analyses adjusted for sex, but no sex-stratified results or interactions were reported The authors noted prior literature on gender differences in CRP associations (e.g., stronger in women), but did not explore sex-based differences in their own findings
John-Henderson et al. [45, 46]	Frequency of thoughts of historical loss	100	Native American adults, ages 20–78 years (Blackfeet Reservation, Montana)	Cross-sectional study	N/A	Obtained by researchers at research office	Greater frequency of thoughts about historical loss predicted higher CRP in a Native American community	Sex was included as a covariate in all statistical models, but no sex-stratified analyses were reported The study noted that historical loss scores were not significantly associated with participant sex in bivariate correlations
Manczak et al. [63]	Dispositional affective empathy, Depressive symptoms	4,603	Adults, ages 24–40 (Nationally representative sample)	Longitudinal cohort study (National Longitudinal Study of Adolescent to Adult Health; Add Health)	8 years (data collected at Wave IV (2008–2009) and Wave V (2016–2018))	Obtained by researchers during in-person assessments	Higher ratings of empathy predicted higher CRP in those with low grade depression; however, no interaction was seen in high grade depression High depressive symptoms independently predicted elevated CRP levels, but they did not amplify the effect of empathy	Sex was included as a covariate in adjusted models, and post hoc tests examined interaction with gender, but no significant moderation by gender was found; results were not disaggregated by sex
McDade et al. [64]	Perceived stress, chronic stress, depressive symptoms, loneliness, perceived social support	188 (subset with CRP measurements)	Middle-aged and older adults, ages 52–70 (Cook County, IL)	Longitudinal study (Chicago Health, Aging, and Social Relations Study)	3 years, with data collected at baseline and two annual follow-ups	Study staff during laboratory visits	Current perceived stress was found to be associated with increased CRP Chronic stress, loneliness, and social support were not significantly predictive of CRP	Sex was included as a covariate in multivariable models; females had significantly higher CRP than males The authors did not test for interaction effects by sex or conduct stratified analyses; however, sex remained a significant predictor of CRP after adjusting for behavioral and psychosocial variables
Shanahan et al. [65]	Depression with co-occurring asthma	1,420	Children/adolescents, ages 10–16 (Western North Carolina)	Longitudinal, cohort study (data from the Great Smoky Mountains Study; GSMS)	6 years	Obtained by researchers during in-person assessments	Co-occurrence of asthma and depression was associated with elevated CRP (CRP > 2 mg/L)	Sex was included as a covariate in all analyses, but no stratified results or interaction tests by sex were reported
Shanahan et al. [66]	Exposure to own or others'suicide attempts during adolescence	7,884	Adolescents/young adults, ages 12–19 years at baseline (Nationally representative sample)	Longitudinal cohort study (National Longitudinal Study of Adolescent to Adult Health; Add Health)	14 years (wave 1: 1994-1995, wave 4: 2007-2008)	Field interviewers during in-home interviews	In males, adolescent suicide attempts (age ≤18 years) and network suicide attempts (friends’ or family members’) during young adulthood were significantly associated with elevated CRP	Sex-stratified models were conducted; adolescent suicide attempts significantly predicted elevated CRP in males but not in females Follow-up analyses confirmed significant sex interactions for both individual and network suicide attempts, highlighting sex-specific pathways in inflammation risk

N/A: Not applicable

## Results

**Fig. 1:**
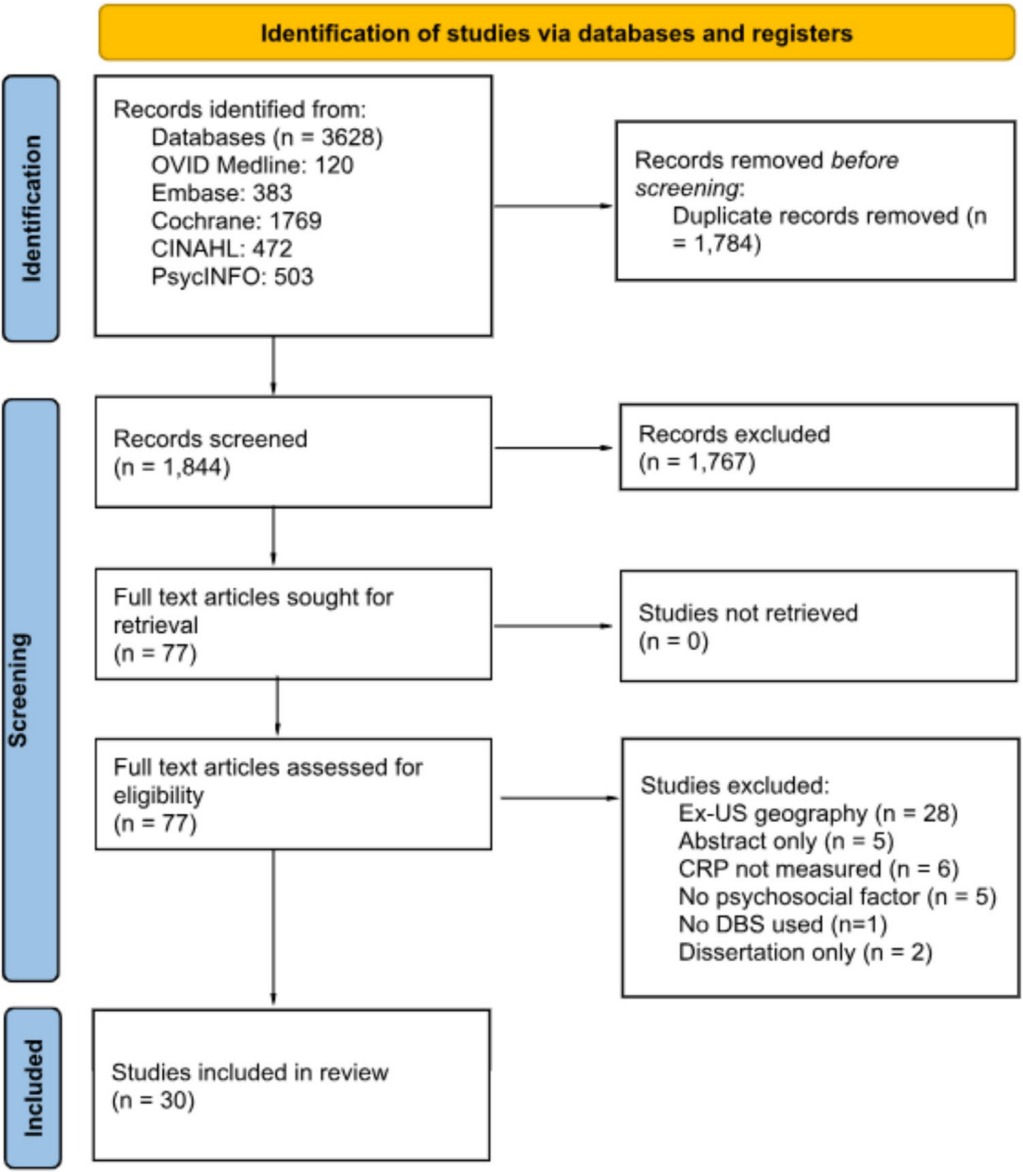
PRISMA chart

The scoping review identified 30 U.S.-based studies using DBS to measure CRP and explore associations with psychosocial factors (Table 1). The PRISMA flowchart (Figure 1) shows the systematic search results.

### Study characteristics

Sample sizes ranged from 20 to 13,236, with a mean of 1,466 (SD: 3,573) and median of 322, reflecting variation in scale—from small, targeted studies to nationally representative surveys. Study designs included cross-sectional (n = 15), longitudinal (n = 14), and experimental (n = 1), with follow-up durations ranging from 3 to 14 years. Participants included children, adolescents, young and older adults, and vulnerable groups such as Native Americans, transgender youth, and immigrant communities. DBS was primarily collected by trained staff during home or lab visits, though some studies used participant self-collection in remote settings. Most studies were nationally representative (32%) or urban-based (29%), with the remainder in rural or regional contexts.

### Associations between psychosocial factors and CRP

To reflect the LCHD framework—findings are organized by major psychosocial domains that represent risk exposures across the life span.

### Socioeconomic and neighborhood contexts

Eight studies explored associations between socioeconomic and neighborhood factors and CRP levels. Boch et al. [37] found that childhood exposure to paternal incarceration increased the odds of low-grade inflammation in adult women (AOR: 1.24–1.48), with no effect in men or for maternal incarceration. Freeman et al. [40] reported higher CRP among individuals with lower perceived social status, particularly in men. Neighborhood characteristics were also linked to CRP. Holmes et al. [41] found residents of high-disorder neighborhoods were 19% more likely to have elevated CRP, while those in areas with greater social capital had a 7% reduced likelihood. Curci et al. [39] showed that prenatal neighborhood disadvantage predicted higher CRP at age 7.5. Schmeer et al. [44] observed dose-response increases in CRP among children exposed to poor physical home environments, with a 62% higher CRP level in those experiencing ≥3 environmental stressors.

### Adverse life events and trauma

Three studies examined adversity-related stressors, including childhood adversity, early life stress (ELS), and suicidality. John-Henderson et al. [45, 46] demonstrated that childhood adversity heightened the inflammatory response to sleep restriction, with significantly elevated CRP in high-adversity individuals. Two studies found no direct link between ELS and CRP in specific populations: Méndez Leal et al. [47] in pregnant women, and Yuan et al. [48] across varying severities of ELS. However, Yuan et al. reported that ELS moderated the relationship between CRP and neural activation during emotion regulation. These findings highlight the importance of moderating contextual factors—such as sex, life stage, and physiological state—in interpreting the impact of early adversity on inflammation.

### Social experiences and interpersonal dynamics

Eight studies assessed the impact of social isolation, discrimination, and relational stress on CRP, finding consistent associations with systemic inflammation. Cudjoe et al. [51] reported that social isolation among Medicare beneficiaries was associated with a 0.13-unit increase in log-transformed CRP, rising to 0.18 with more severe isolation. Goosby et al. [53] found that perceived discrimination among low-income African American youth was linked to a 0.34 standard deviation increase in log CRP, even after controlling for demographic and anthropometric factors. Bullying showed particularly striking associations. Copeland et al. [50] identified a dose-response relationship between cumulative bullying victimization and elevated adult CRP. Interestingly, individuals who engaged in bullying had lower CRP levels, suggesting a possible stress-buffering effect of perceived social dominance.

### Mental health and emotional well-being

Eleven studies explored how mental health and psychological factors influence CRP. Shanahan et al. [66] found that adolescent suicide attempts predicted higher CRP in males, with similar associations observed for network suicidality—suicide attempts by friends or family members—in young adulthood. These associations were present in males but not females, suggesting sex-based differences in how adversity relates to inflammation. Ironson et al. [62] reported that greater life satisfaction and positive affect were linked to lower CRP in a nationally representative adult sample. Conversely, depressive symptoms—particularly when co-occurring with chronic conditions like asthma—were associated with elevated CRP. Shanahan et al. [65] found that the interaction between asthma and depression significantly increased the likelihood of CRP exceeding 3 mg/L. Other studies identified more nuanced effects. Manczak et al. [63] reported that empathy was associated with higher CRP in individuals with low-grade depression, but not in those with more severe symptoms, suggesting that traits like empathy may have context-dependent effects on inflammation.

### Consideration of sex and gender in CRP associations

Only five studies (16.7%) conducted fully stratified analyses by sex or gender identity; most treated sex as a control variable. Certain studies reported sex-specific findings. Boch et al. (2015) and Shanahan et al. [66] found significant associations between psychosocial stressors and CRP in females and males, respectively, with null results in the other group. Freeman et al. [40] identified an interaction between subjective social status and sex, significant only in men. Gender identity was rarely analyzed as a distinct factor, though McQuillan et al. [56] compared CRP levels among transfeminine and transmasculine youth. Overall, findings reflect inconsistent integration of sex and gender and underscore the need for more disaggregated analyses in future research.

### Clinical significance of associations

Many studies demonstrated clinically significant associations between psychosocial factors and CRP. For example, childhood exposure to paternal incarceration increased the odds of low-grade inflammation (CRP 3–10 mg/L) by up to 48% in women (Boch et al. 2015). Severe social isolation among older adults was associated with a 0.18-unit increase in log CRP [51]. Children exposed to home stressors such as clutter or uncleanliness had 11% higher CRP per stressor, with increases reaching 62% in the most affected cases [44]. These findings suggest psychosocial exposures are associated with measurable CRP increases that may contribute to long-term health risks.

## Discussion

### Foundational studies

CRP is a well-established biomarker of systemic inflammation and an independent predictor of CVD risk [67, 68]. The central role of inflammation in chronic disease has made CRP a widely used marker in psychosocial and population health research. Venous blood studies consistently link psychosocial stressors—including socioeconomic disadvantage, early life adversity, and mental health conditions—to elevated CRP, laying the groundwork for DBS-based research (Alley et al. 2006, [3], Ranjit et al. 2007; [69], [70], [71].

Large-scale U.S. cohorts reveal clear socioeconomic gradients in CRP, with higher levels among individuals with lower income or education (Alley et al. 2006; [3], Ranjit et al. 2007), and similar patterns among children exposed to poverty and other household stressors [69]. These associations align with stress biology frameworks, in which chronic HPA axis and sympathetic activation from prolonged stress increase cytokine production and CRP release [6], [4].

Venous studies support these mechanisms across multiple domains. Dalechek et al. [70] found higher CRP among adults with adverse childhood experiences (ACEs), and Deighton et al. [72] showed early adversity alters immune function through neuroendocrine dysregulation. Neighborhood-level factors also appear to shape inflammatory profiles: children exposed to poverty and crime [73] and adults in disadvantaged settings [74] had elevated CRP—supporting the LCHD framework that social conditions embed biologically over time.

Mental health is similarly implicated. Ji et al. [75] found depressive symptoms were associated with higher CRP, consistent with meta-analytic data showing ~27% of depressed individuals exhibit low-grade inflammation [71]. Additional traits such as loneliness, low positive affect, and pessimism have similarly been linked to CRP elevation [76], [77]. Importantly, some studies suggest bidirectional relationships: Das [78] found elevated CRP predicted future depression and stress, indicating CRP may function as both marker and mediator of psychosocial burden.

Finally, sex-based differences in the relationship between psychosocial stress and CRP have been reported. For example, Lockwood et al. [7] observed that depression-inflammation associations were stronger in men than women, possibly reflecting both biological mechanisms—such as hormonal modulation of inflammatory signaling—and social-contextual factors that shape stress exposure and coping responses.

Collectively, venous blood studies provide strong empirical and mechanistic support for psychosocial–CRP associations, forming a foundation for expanding this research through DBS in more inclusive and scalable settings.

### DBS fidelity

A high-sensitivity CRP assay for DBS has been developed and validated [79] and widely applied in population studies. Lab-based comparisons show strong agreement between DBS and venous blood [29, 79–81]. Field studies also report reasonably strong correlation coefficients (r) between DBS and matching venous blood values when collected by trained study staff: 0.82 (n = 395, [82], 0.84 (n = 395, [83], and 0.87 (n = 396, [30]. Reed et al. [29] further validated home-based, supervised DBS collection via videochat, with a correlation of r = 0.75 (n = 41) compared to venous samples, and an intra-class correlation of 0.74 between self-collected and lab-collected DBS samples taken 4–5 days apart. Studies have found that small spot sizes and short drying times reduced CRP accuracy, while humidity and temperature had no major effect [84, 85]. Overall, DBS validation studies show good agreement with gold standard venipuncture, though further field validation is warranted [86], [29], [33].

Schakelaar et al. [87] assessed performance across the CRP range, reporting strong correlation with venous samples (R^2^ = 0.986) and high precision at higher CRP levels (>9 mg/L). Although variability increased at very low CRP levels, accuracy remained within clinically acceptable margins, supporting DBS use in most research and diagnostic settings.

Future assay development and validation for hs-CRP assays may investigate the added value of standardized sample collection protocols and novel DBS collection devices, such as the DBS Capitainer qDBS [88], HemaSpot devices [49], Tasso M20 [89], or Drawbridge OneDraw [90] collection devices. The latter two offer greater ease and reproducibility for in-home use over Capitainer and may improve hs-CRP reliability in field studies [88], [49], [89], [90].

### What DBS studies add to psychosocial inflammatory biomarker research

DBS-based studies extended foundational venous blood research by enabling the study of nuanced psychosocial stressors in historically underrepresented populations.

### Novel psychosocial contexts

DBS has facilitated the examination of stressors that are often difficult to capture using venous blood collection, particularly in studies requiring flexibility in setting, timing, and staffing. Guardino et al. [54] used home visits to assess financial stress, discrimination, and violence among postpartum women, overcoming barriers like transportation and caregiving. McQuillan et al. [56] collected samples from transgender and gender-nonconforming youth in affirming community settings, avoiding clinic-based discomfort. John-Henderson et al. [45, 46] studied historical loss among Native American adults, collecting samples on tribal lands to reduce disruption and respect cultural norms. In each case, the ability to incorporate biomarker collection into nontraditional, participant-centered contexts was essential for conducting research with these populations on their unique psychosocial stressors.

### Longitudinal study designs

DBS is particularly well-suited for longitudinal study designs that involve repeated sampling over weeks, months, or years due to its low cost, minimal invasiveness, and ability to be collected in nonclinical settings [29, 86]. Unlike venipuncture, DBS can be self-collected or administered by nonclinical staff, mailed without cold-chain requirements, and stored at ambient temperatures—reducing participant burden and enhancing follow-up and retention [83, 86]. This advantage is reflected in several studies. For instance, Boch et al. (2015) used DBS over four waves across 14 years to link childhood exposures to adult CRP. Similarly, Curci et al. [39] followed mother-child dyads for 7.5 years, measuring CRP to assess the lasting influence of prenatal neighborhood disadvantage. These studies underscore how DBS enables life-course tracking of inflammation across extended follow-up periods. Although venipuncture can also support repeated measurement—especially in short-term or intensive protocols—DBS offers distinct advantages for long-term (e.g., multi-year) epidemiologic research, particularly in decentralized or resource-limited settings where repeated clinic visits may not be feasible.

### Sampling advantages of DBS research

DBS enables scalable, cost-effective biological sampling across large, diverse cohorts. Its minimally invasive collection, small blood volume requirement, and ability to be used without trained phlebotomists make it well-suited for geographically, socioeconomically, and demographically diverse populations [28]. Participants may also be more willing to provide DBS samples than venous blood [30], improving participation and enhancing generalizability by facilitating inclusion of hard-to-reach groups. For example, Freeman et al. [40] used DBS to examine subjective socioeconomic status and CRP. Over 13,000 respondents provided samples during in-home interviews, yielding a 94% participation rate—exceeding typical rates for venous collection in population surveys. This approach eliminated the need for clinic visits, streamlining data collection while preserving a large, nationally representative sample. Similarly, McDade et al. [42] used DBS to study socioeconomic factors and CRP levels among more than 3,000 older adults, achieving 68% participation, including individuals with physical or cognitive impairments who may have been difficult to sample in clinical settings. These studies demonstrate how DBS facilitates the integration of inflammatory biomarkers into broad, community-based surveys—often in ways that would not be feasible with venous blood collection.

### Underrepresented populations

DBS facilitates research among historically underrepresented groups by lowering logistical and financial barriers while maintaining methodological rigor. Its stability at ambient temperatures supports use in low-resource and remote settings [81], and finger-stick collection allows for field- or home-based sampling without trained phlebotomists. The direct cost of DBS supplies is approximately $5 per participant, compared to $88 for standard outpatient phlebotomy and processing [91], making it especially valuable for pediatric, socioeconomically disadvantaged, rural, or resource-constrained populations.

In addition to logistical benefits, DBS often aligns with participant preferences. In focus groups, low-income urban women favored in-home finger-stick collection by trusted community-based interviewers over clinic visits [31], helping explain the high participation rates by underrepresented populations in DBS-based studies. For example, Holmes et al. (2012) collected samples from foreign-born Brazilian adults in their homes to overcome language and documentation barriers. Guardino et al. [54] reached postpartum women in five U.S. cities using home-based DBS, enrolling a sample that was 77% women of color and 70% living at or below the poverty line. Schmeer et al. [44] gathered inflammation data from children in child-friendly settings, achieving representation from low-income, Hispanic, and immigrant families. Goosby et al. [53] sampled African American adolescents in community settings to study the inflammatory effects of discrimination. Together, these studies demonstrate how DBS enables inclusive, community-based biomarker research with populations for whom venipuncture would likely pose participation barriers—advancing efforts to study health disparities in real-world, participant-centered contexts.

#### Limitations

### Limitations of DBS methodology

Despite its advantages for population-based research, DBS sampling has important limitations. First, the range of validated assays for DBS is narrower than for venous blood, particularly for low-abundance biomarkers requiring high analytical sensitivity [33]. While hs-CRP shows strong agreement with venous samples in both lab and field settings [79], [80], [29], other inflammatory or cardiometabolic markers may exhibit greater variability or lack validation under challenging field conditions.

Second, DBS utilizes capillary rather than venous blood, which may introduce variability due to differences in cellular composition, hematocrit, and peripheral circulation dynamics [92]. These physiological differences may affect analyte concentrations and require correction during analysis. DBS also cannot support immune cell phenotyping (e.g., monocyte subsets or regulatory T cells) or ex vivo stimulation protocols, limiting the ability to assess inflammatory dynamics [93], [87].

Finally, DBS is sensitive to preanalytical conditions—including spot size, drying time, humidity, and temperature during transport and storage—which can impact data quality, especially for field-based or self-collected samples [84, 85]. Standardizing collection protocols and expanding assay validation efforts are needed to improve precision and reproducibility in epidemiologic research [33]. While DBS supports inclusive, scalable study designs, its methodological constraints must be considered when interpreting results or designing studies requiring cellular or functional immune assessments.

### Limitations of this scoping review

This review has several limitations. First, its focus on U.S.-based studies limits generalizability to other cultural and socioeconomic contexts, where psychosocial stressors and their inflammatory effects may vary due to differences in social structures, healthcare systems, and cultural norms [94]. Future research should examine these associations in globally representative settings.

Second, most included studies were observational, limiting causal inference—particularly in cross-sectional designs. Although some used longitudinal methods, confounding from health status and lifestyle behaviors remains a challenge. Robust longitudinal designs are needed to clarify causal pathways.

Third, broad categorization of psychosocial factors (e.g., SES, life adversity) may obscure the complex, overlapping nature of psychosocial stressors. Stressors like economic precarity, discrimination, and mental health conditions often co-occur and may amplify each other and exert distinct biological effects, even when activating similar inflammatory pathways. More granular categorization of psychosocial factors is needed to capture these nuances.

Fourth, while we extracted data on sex and gender analyses, few studies conducted fully stratified models. This limited our ability to assess sex-specific patterns in associations. More deliberate integration of sex and gender analyses is needed to better capture biological and contextual heterogeneity in inflammatory responses.

Finally, reliance on self-reported psychosocial measures introduces potential for mood and memory bias [95]. Notably, studies of ACEs suggest that subjective recall can have stronger associations with health outcomes than objective records [96]. Future research should integrate subjective and objective measures alongside physiological stress markers to better characterize these relationships.

## Conclusion

This scoping review highlights significant associations between psychosocial factors—such as socioeconomic conditions, life adversity, social relationships, and emotional well-being—and systemic inflammation. DBS emerges as a valuable tool for large-scale biomarker research, particularly for underrepresented groups and resource-limited settings. While many studies accounted for gender- and sex-based differences, few conducted fully stratified analyses, suggesting an area for future exploration. As DBS assay accuracy and precision continue to improve, future research should leverage this methodology to explore a broader range of psychosocial and structural factors, such as generational trauma linked to global patterns of immigration, displacement, and war, and investigate additional validated inflammatory biomarkers, such as IL-6 and TNF-α [32], [33].

## Supplementary Information

Below is the link to the electronic supplementary material.

Supplementary file1 (DOCX 23 kb)

## Data Availability

The data from studies included in our scoping review are publicly available online. Data collected for our review is provided within the manuscript and supplementary information files.
