# Supplementary material for: Investigating psychosocial factors and systemic inflammation using dried blood spots: a scoping review
Source: Soc Psychiatry Psychiatr Epidemiol. 2025 Jun 13;60(11):2533–52. doi: 10.1007/s00127-025-02941-0 (PMC12572048; doi:10.1007/s00127-025-02941-0)
Supplement: Supplementary file 1 — Supplementary file1 (DOCX 23 kb) [file 127_2025_2941_MOESM1_ESM.docx]

## **Investigating psychosocial factors and systemic inflammation using dried blood spots: a scoping review**

## *Online Supplemental Files*

## Database Search Overview

| Database | Coverage | Date Searched | Results |
| --- | --- | --- | --- |
| Ovid MEDLINE(R) and Epub Ahead of Print, In-Process, In-Data-Review & Other Non-Indexed Citations, Daily and Versions(R) | 1946 to present | 04/24/2024 | 120 |
| Cochrane Database of Systematic Reviews Issue 2 of 12, February 2022 (Wiley) | 1995 to present | 04/24/2024 | 21 |
| Cochrane Central Register of Controlled Trials Issue 2 of 12, February 2022  (Wiley) | 1995 to present | 04/24/2024 | 1748 |
| Embase (Elsevier) | 1947 to present | 04/24/2024 | 383 |
| CINAHL Plus with Full Text (EBSCOhost) | 1937 to present | 04/24/2024 | 472 |
| PsycInfo (EBSCOhost) | 1800s to present | 04/24/2024 | 503 |
| Total | |  | 3628 |
| Total After De-Duplication | |  | 1844 |

We searched the databases listed above on **April 24, 2024.** Records from each database were exported to EndNote. Duplicates were removed through the “Find Duplicates” function within EndNote and were manually reviewed. Search strategies from each of the bibliographic databases are available below:

# Ovid MEDLINE(R)

Ovid MEDLINE(R) ALL <1946 to April 23, 2024>

1 exp Dried Blood Spot Testing/ 2143

2 dbs.ti. 1120

3 ("Guthrie card*" or "Guthrie paper*" or "dried blood" or "dried whole blood spot*" or "dried whole bloodspot*").ti,ab. 7355

4 (("blood blot*" or "blood spot*" or bloodspot*) and "filter paper*").ti,ab. 1061

5 (("blood blot*" or "blood spot*" or bloodspot* or "finger-stick blood") adj3 (collect* or sampl*)).ti,ab. 2376

6 1 or 2 or 3 or 4 or 5 9400

7 exp Blood Proteins/ 1799531

8 exp Biomarkers/ 901889

9 exp *Enzymes/ 2014871

10 exp *Peptides/ 1634871

11 exp *Proteins/ 5058541

12 exp *Hormones/ 927079

13 exp *Antigens/ 615458

14 exp *Antibodies/ 448713

15 exp *Genomics/ 76080

16 exp Epigenomics/ 8527

17 exp RNA, Messenger/ 452003

18 exp *Lipids/ 751248

19 (lipid* or marker*).ti. 315118

20 (biomarker* or antibod* or antigen* or apolipoprotein* or "biological marker*" or "DNA methylation" or "dna sequenc*" or enzyme* or epigenetic* or "gene expression*" or "genetic marker*" or hormon* or mediator* or mRNA* or peptide* or protein*).ti,ab. 6996455

21 7 or 8 or 9 or 10 or 11 or 12 or 13 or 14 or 15 or 16 or 17 or 18 or 19 or 20 10667473

22 exp *Community Health Centers/ 7664

23 exp *Community Mental Health Centers/ 2173

24 exp *Correctional Facilities/ 7613

25 *Public Health/ 58014

26 *Population Health/ 1710

27 exp *Rehabilitation Centers/ 7544

28 exp *Residential Facilities/ 39063

29 (place or "health centre*" or "health center*" or incarcerat* or neighborhood* or neighbourhood* or "nursing home*" or penitentiar* or prison* or "rehabilitation center*" or "rehabilitation centre*" or "treatment center*" or "treatment centre*").ti. 84815

30 (("assisted living" or home or homes or house or household* or houses or community or facilit* or off-site or off-sites or "population-base*") and (setting* or location*)).ti. 6950

31 22 or 23 or 24 or 25 or 26 or 27 or 28 or 29 or 30 189641

32 exp Cardiovascular Diseases/ 2782311

33 exp Stroke/ 181070

34 exp *Dyslipidemias/ 61816

35 exp Hyperlipidemias/ 71576

36 *Inflammation/ 82163

37 ((cardiac or cardiovascular or heart or aortic) adj2 (disease* or disorder or disorders or malformation* or dysfunction*)).ti,ab. 481937

38 (inflammat* adj2 (heart or cardiac)).ti,ab. 4187

39 inflammation*.ti. 117065

40 ("acute coronary syndrome" or "angina pectoris" or asystole* or atherosclero* or atherosclerotic or "cardiac arrest*" or "cardiac infarct*" or "cardiomyopath*" or "cardiovascular abnormal*" or "cardiovascular event*" or "cardiovascular infection*" or "cardiovascular outcome*" or "cerebral vascular accident*" or "cerebrovascular accident*" or "high cholesterol " or "coronary artery disease*" or "coronary artery obstruction*" or "coronary artery thrombos*" or "coronary occlusion" or endocarditis or "heart aneurysm*" or "heart arrest*" or "heart attack*" or "heart failure" or "heart infarct*" or "high blood pressure" or hypertension or hyperlipidemia or "low grade inflammation" or dyslipidemia or "myocardial failure*" or "myocardial infarct*" or "myocardial ischemia" or "myocardium failure " or "myocardium infarct*" or "percutaneous coronary intervention*" or pericarditis or "peripheral arterial disease" or stenocardia* or stroke* or "subclinical cardiovascular" or "vascular malformation*" or "venous insufficien*" or "ventricular dysfunction*" or "ventricular outflow obstruction*").ti,ab. 1584009

41 32 or 33 or 34 or 35 or 36 or 37 or 38 or 39 or 40 3548279

42 6 and 21 and 41 340

43 21 and 31 and 41 653

44 42 or 43 993

45 limit 44 to yr="2022 -Current" 120

Cochrane Library

Cochrane Database of Systematic Reviews

Issue 4 of 12, April 2024

Cochrane Central Register of Controlled Trials

Issue 3 of 12, March 2024

Search Name:

Date Run: 24/04/2024 16:34:21

Comment:

ID Search Hits

#1 MeSH descriptor: [Dried Blood Spot Testing] explode all trees 38

#2 ("DBS"):ti 278

#3 (("Guthrie card" OR "Guthrie paper" OR "dried blood" OR "dried whole blood spot" OR "dried whole bloodspot")):ti,ab,kw 557

#4 ((("blood blot" or "blood spot" or bloodspot* or "finger-stick blood") Near/3 (collect* or sampl*))):ti,ab,kw 170

#5 ((("blood blot" OR "blood spot" OR bloodspot*) AND ("filter paper"))):ti,ab,kw 20

#6 #1 OR #2 OR #3 OR #4 OR #5 894

#7 MeSH descriptor: [Blood Proteins] explode all trees 76011

#8 MeSH descriptor: [Biomarkers] explode all trees 31343

#9 MeSH descriptor: [Enzymes] explode all trees 43934

#10 MeSH descriptor: [Peptides] explode all trees 99699

#11 MeSH descriptor: [Proteins] explode all trees 170467

#12 MeSH descriptor: [Hormones] explode all trees 79187

#13 MeSH descriptor: [Antigens] explode all trees 21009

#14 MeSH descriptor: [Antibodies] explode all trees 37511

#15 MeSH descriptor: [Genome] explode all trees 3323

#16 MeSH descriptor: [Epigenomics] explode all trees 28

#17 MeSH descriptor: [RNA, Messenger] explode all trees 1853

#18 MeSH descriptor: [Lipids] explode all trees 64366

#19 (lipid* OR marker*):ti 22109

#20 ((biomarker* or antibod* or antigen* or apolipoprotein* or "biological marker" or "DNA methylation" or "dna sequenc" or enzyme* or epigenetic* or "gene expression" or "genetic marker" or hormon* or mediator* or mRNA* or peptide* or protein*)):ti,ab,kw 327456

#21 #7 OR #8 OR #9 OR #10 OR #11 OR #12 OR #13 OR #14 OR #15 OR #16 OR #17 OR #18 OR #19 288855

#22 MeSH descriptor: [Community Health Centers] explode all trees 718

#23 MeSH descriptor: [Community Mental Health Centers] explode all trees 158

#24 MeSH descriptor: [Correctional Facilities] explode all trees 209

#25 MeSH descriptor: [Public Health] explode all trees 634194

#26 MeSH descriptor: [Population Health] explode all trees 1064

#27 MeSH descriptor: [Rehabilitation Centers] explode all trees 813

#28 MeSH descriptor: [Residential Facilities] explode all trees 2611

#29 ((place or "health centre" or "health center" or incarcerat* or neighborhood* or neighbourhood* or "nursing home" or penitentiar* or prison* or "rehabilitation center" or "rehabilitation centre" or "treatment center" or "treatment centre")):ti,ab,kw 42792

#30 ((("assisted living" or home or homes or house or household* or houses or community or facilit* or off-site or off-sites or "population-base") and (setting* or location*))):ti,ab,kw 32621

#31 #22 OR #23 OR #24 OR #25 OR #26 OR #27 OR #28 OR #29 OR #30 681366

#32 MeSH descriptor: [Cardiovascular Diseases] explode all trees 155445

#33 MeSH descriptor: [Stroke] explode all trees 17328

#34 MeSH descriptor: [Dyslipidemias] explode all trees 9583

#35 MeSH descriptor: [Hyperlipidemias] explode all trees 7989

#36 MeSH descriptor: [Inflammation] explode all trees 16387

#37 (((cardiac or cardiovascular or heart or aortic) Near/2 (disease* or disorder or disorders or malformation* or dysfunction*))):ti,ab,kw 68598

#38 ((inflammat* Near/2 (heart or cardiac))):ti,ab,kw 472

#39 (inflammation):ti 8579

#40 (("acute coronary syndrome" or "angina pectoris" or asystole* or atherosclero* or atherosclerotic or "cardiac arrest” or "cardiac infarct” or "cardiomyopath” or "cardiovascular abnormal” or "cardiovascular event” or "cardiovascular infection” or "cardiovascular outcome” or "cerebral vascular accident” or "cerebrovascular accident” or "high cholesterol " or "coronary artery disease” or "coronary artery obstruction” or "coronary artery thrombos” or "coronary occlusion" or endocarditis or "heart aneurysm” or "heart arrest” or "heart attack” or "heart failure" or "heart infarct” or "high blood pressure" or hypertension or hyperlipidemia or "low grade inflammation" or dyslipidemia or "myocardial failure” or "myocardial infarct” or "myocardial ischemia" or "myocardium failure " or "myocardium infarct” or "percutaneous coronary intervention” or pericarditis or "peripheral arterial disease" or stenocardia* or stroke* or "subclinical cardiovascular" or "vascular malformation” or "venous insufficien” or "ventricular dysfunction” or "ventricular outflow obstruction”)):ti,ab,kw 227528

#41 #32 or #33 or #34 or #35 or #36 #38 or #39 or #40 294508

#42 #6 AND #21 AND #41 14

#43 #21 AND #31 AND #41 39808

#44 #42 OR #43 39813

#45 #44 with Cochrane Library publication date Between Feb 2022 and Apr 2024, in Cochrane Reviews, Trials 1769

# Embase( Elsevier)

Embase

Session Results

.......................................................

No. Query Results Results Date

#43. #42 AND (2022:py OR 2023:py OR 2024:py) 383 25 Apr 2024

#42. #40 OR #41 2,350 25 Apr 2024

#41. #20 AND #30 AND #39 1,474 25 Apr 2024

#40. #6 AND #20 AND #39 878 25 Apr 2024

#39. #31 OR #32 OR #33 OR #34 OR #35 OR #36 OR #37 OR 4,742,658 25 Apr 2024

#38

#38. 'acute coronary syndrome':ab,ti OR 'angina 2,473,716 25 Apr 2024

pectoris':ab,ti OR asystole*:ab,ti OR

atherosclero*:ab,ti OR atherosclerotic:ab,ti OR

'cardiac arrest*':ab,ti OR 'cardiac

infarct*':ab,ti OR 'cardiomyopath*':ab,ti OR

'cardiovascular abnormal*':ab,ti OR

'cardiovascular event*':ab,ti OR 'cardiovascular

infection*':ab,ti OR 'cardiovascular

outcome*':ab,ti OR 'cerebral vascular

accident*':ab,ti OR 'cerebrovascular

accident*':ab,ti OR 'high cholesterol':ab,ti OR

'coronary artery disease*':ab,ti OR 'coronary

artery obstruction*':ab,ti OR 'coronary artery

thrombos*':ab,ti OR 'coronary occlusion':ab,ti OR

endocarditis:ab,ti OR 'heart aneurysm*':ab,ti OR

'heart arrest*':ab,ti OR 'heart attack*':ab,ti OR

'heart failure':ab,ti OR 'heart infarct*':ab,ti

OR 'high blood pressure':ab,ti OR

hypertension:ab,ti OR hyperlipidemia:ab,ti OR

'low grade inflammation':ab,ti OR

dyslipidemia:ab,ti OR 'myocardial failure*':ab,ti

OR 'myocardial infarct*':ab,ti OR 'myocardial

ischemia':ab,ti OR 'myocardium failure':ab,ti OR

'myocardium infarct*':ab,ti OR 'percutaneous

coronary intervention*':ab,ti OR

pericarditis:ab,ti OR 'peripheral arterial

disease':ab,ti OR stenocardia*:ab,ti OR

stroke*:ab,ti OR 'subclinical

cardiovascular':ab,ti OR 'vascular

malformation*':ab,ti OR 'venous

insufficien*':ab,ti OR 'ventricular

dysfunction*':ab,ti OR 'ventricular outflow

obstruction*':ab,ti

#37. inflammation*.ti 82 25 Apr 2024

#36. (inflammat* NEAR/2 (heart OR cardiac)):ab,ti 6,224 25 Apr 2024

#35. ((cardiac OR cardiovascular OR heart OR aortic) 725,476 25 Apr 2024

NEAR/2 (disease* OR disorder OR disorders OR

malformation* OR dysfunction*)):ab,ti

#34. 'inflammation'/mj 147,863 25 Apr 2024

#33. 'hyperlipidemia'/exp/mj 62,073 25 Apr 2024

#32. 'dyslipidemia'/exp/mj 15,502 25 Apr 2024

#31. 'cardiovascular disease'/exp/mj 3,525,487 25 Apr 2024

#30. #21 OR #22 OR #23 OR #24 OR #25 OR #26 OR #27 OR 239,729 25 Apr 2024

#28 OR #29

#29. ('assisted living':ti OR home:ti OR homes:ti OR 9,507 25 Apr 2024

house:ti OR household*:ti OR houses:ti OR

community:ti OR facilit*:ti OR 'off site':ti OR

'off sites':ti OR 'population-base*':ti) AND

(setting*:ti OR location*:ti)

#28. place:ti OR 'health centre*':ti OR 'health 103,057 25 Apr 2024

center*':ti OR incarcerat*:ti OR neighborhood*:ti

OR neighbourhood*:ti OR 'nursing home*':ti OR

penitentiar*:ti OR prison*:ti OR 'rehabilitation

center*':ti OR 'rehabilitation centre*':ti OR

'treatment center*':ti OR 'treatment centre*':ti

#27. 'population health'/exp/mj 2,331 25 Apr 2024

#26. 'public health'/mj 86,288 25 Apr 2024

#25. 'correctional facility'/exp/mj 9,333 25 Apr 2024

#24. 'residential home'/mj 3,175 25 Apr 2024

#23. 'rehabilitation center'/mj 4,733 25 Apr 2024

#22. 'community mental health center'/exp/mj 2,146 25 Apr 2024

#21. 'health care facility'/mj 29,057 25 Apr 2024

#20. #7 OR #8 OR #9 OR #10 OR #11 OR #12 OR #13 OR #14 13,224,959 25 Apr 2024

OR #15 OR #16 OR #17 OR #18 OR #19

#19. biomarker*:ab,ti OR antibod*:ab,ti OR 8,909,402 25 Apr 2024

antigen*:ab,ti OR apolipoprotein*:ab,ti OR

'biological marker*':ab,ti OR 'dna

methylation':ab,ti OR 'dna sequenc*':ab,ti OR

enzyme*:ab,ti OR epigenetic*:ab,ti OR 'gene

expression*':ab,ti OR 'genetic marker*':ab,ti OR

hormon*:ab,ti OR mediator*:ab,ti OR mrna*:ab,ti

OR peptide*:ab,ti OR protein*:ab,ti

#18. lipid*:ti OR marker*:ti 406,814 25 Apr 2024

#17. 'lipid'/exp/mj 833,996 25 Apr 2024

#16. 'messenger rna'/exp/mj 84,076 25 Apr 2024

#15. 'epigenetics'/mj 35,422 25 Apr 2024

#14. 'genomics'/exp/mj 54,239 25 Apr 2024

#13. 'antibody'/exp/mj 601,119 25 Apr 2024

#12. 'antigen'/exp/mj 695,280 25 Apr 2024

#11. 'hormone'/exp/mj 26,572 25 Apr 2024

#10. 'peptides and proteins'/exp/mj 7,316,202 25 Apr 2024

#9. 'enzyme'/exp/mj 2,222,822 25 Apr 2024

#8. 'biological marker'/exp 469,560 25 Apr 2024

#7. 'plasma protein'/exp 1,912,434 25 Apr 2024

#6. #1 OR #2 OR #3 OR #4 OR #5 16,934 25 Apr 2024

#5. ('blood blot*':ab,ti OR 'blood spot*':ab,ti OR 1,535 25 Apr 2024

bloodspot*:ab,ti) AND 'filter paper*':ab,ti

#4. (('blood blot*' OR 'blood spot*' OR bloodspot* OR 3,536 25 Apr 2024

'finger-stick blood') NEAR/3 (collect* OR

sampl*)):ab,ti

#3. 'guthrie card*':ab,ti OR 'guthrie paper*':ab,ti 11,073 25 Apr 2024

OR 'dried blood':ab,ti OR 'dried whole blood

spot*':ab,ti OR 'dried whole bloodspot*':ab,ti

#2. dbs:ti 3,241 25 Apr 2024

#1. 'dried blood spot testing'/exp 6,814 25 Apr 2024

.......................................................#1

'dried blood spot testing'/exp

# CINAHL (EBSCO)

Wednesday, April 24, 2024 7:50:35 PM

# Query Limiters/Expanders Last Run Via Results

S42 S40 OR S41 Expanders - Apply equivalent subjects

Search modes - Boolean/Phrase Interface - EBSCOhost Research Databases

Search Screen - Advanced Search

Database - CINAHL Plus with Full Text 472

S41 S21 AND S30 AND S39 Expanders - Apply equivalent subjects

Search modes - Boolean/Phrase Interface - EBSCOhost Research Databases

Search Screen - Advanced Search

Database - CINAHL Plus with Full Text 304

S40 S6 AND S21 AND S39 Expanders - Apply equivalent subjects

Search modes - Boolean/Phrase Interface - EBSCOhost Research Databases

Search Screen - Advanced Search

Database - CINAHL Plus with Full Text 168

S39 S31 OR S32 OR S33 OR S34 OR S35 OR S36 OR S37 OR S38 Expanders - Apply equivalent subjects

Search modes - Boolean/Phrase Interface - EBSCOhost Research Databases

Search Screen - Advanced Search

Database - CINAHL Plus with Full Text 880,220

S38 TI ( "acute coronary syndrome" or "angina pectoris" or asystole* or atherosclero* or atherosclerotic or "cardiac arrest*" or "cardiac infarct*" or "cardiomyopath*" or "cardiovascular abnormal*" or "cardiovascular event*" or "cardiovascular infection*" or "cardiovascular outcome*" or "cerebral vascular accident*" or "cerebrovascular accident*" or "high cholesterol " or "coronary artery disease*" or "coronary artery obstruction*" or "coronary artery thrombos*" or "coronary occlusion" or endocarditis or "heart aneurysm*" or "heart arrest*" or "heart attack*" or "heart failure" or "heart infarct*" or "high blood pressure" or hypertension or hyperlipidemia or "low grade inflammation" or dyslipidemia or "myocardial failure*" or "myocardial infarct*" or "myocardial ischemia" or "myocardium failure " or "myocardium infarct*" or "percutaneous coronary intervention*" or pericarditis or "peripheral arterial disease" or stenocardia* or stroke* or "subclinical cardiovascular" or "vascular malformation*" or "venous insufficien*" or "ventricular dysfunction*" or "ventricular outflow obstruction*" ) OR AB ( "acute coronary syndrome" or "angina pectoris" or asystole* or atherosclero* or atherosclerotic or "cardiac arrest*" or "cardiac infarct*" or "cardiomyopath*" or "cardiovascular abnormal*" or "cardiovascular event*" or "cardiovascular infection*" or "cardiovascular outcome*" or "cerebral vascular accident*" or "cerebrovascular accident*" or "high cholesterol " or "coronary artery disease*" or "coronary artery obstruction*" or "coronary artery thrombos*" or "coronary occlusion" or endocarditis or "heart aneurysm*" or "heart arrest*" or "heart attack*" or "heart failure" or "heart infarct*" or "high blood pressure" or hypertension or hyperlipidemia or "low grade inflammation" or dyslipidemia or "myocardial failure*" or "myocardial infarct*" or "myocardial ischemia" or "myocardium failure " or "myocardium infarct*" or "percutaneous coronary intervention*" or pericarditis or "peripheral arterial disease" or stenocardia* or stroke* or "subclinical cardiovascular" or "vascular malformation*" or "venous insufficien*" or "ventricular dysfunction*" or "ventricular outflow obstruction*" ) Expanders - Apply equivalent subjects

Search modes - Boolean/Phrase Interface - EBSCOhost Research Databases

Search Screen - Advanced Search

Database - CINAHL Plus with Full Text 419,230

S37 TI inflammation* Expanders - Apply equivalent subjects

Search modes - Boolean/Phrase Interface - EBSCOhost Research Databases

Search Screen - Advanced Search

Database - CINAHL Plus with Full Text 21,262

S36 TI ( inflammat* N2 (heart or cardiac) ) OR AB ( inflammat* N2 (heart or cardiac) ) Expanders - Apply equivalent subjects

Search modes - Boolean/Phrase Interface - EBSCOhost Research Databases

Search Screen - Advanced Search

Database - CINAHL Plus with Full Text 1,144

S35 TI (( cardiac or cardiovascular or heart or aortic) N2 (disease* or disorder or disorders or malformation* or dysfunction* )) OR AB (( cardiac or cardiovascular or heart or aortic) N2 (disease* or disorder or disorders or malformation* or dysfunction* )) Expanders - Apply equivalent subjects

Search modes - Boolean/Phrase Interface - EBSCOhost Research Databases

Search Screen - Advanced Search

Database - CINAHL Plus with Full Text 123,954

S34 (MM "Inflammation") Expanders - Apply equivalent subjects

Search modes - Boolean/Phrase Interface - EBSCOhost Research Databases

Search Screen - Advanced Search

Database - CINAHL Plus with Full Text 21,445

S33 (MM "Hyperlipidemia") Expanders - Apply equivalent subjects

Search modes - Boolean/Phrase Interface - EBSCOhost Research Databases

Search Screen - Advanced Search

Database - CINAHL Plus with Full Text 7,717

S32 (MH "Stroke+") Expanders - Apply equivalent subjects

Search modes - Boolean/Phrase Interface - EBSCOhost Research Databases

Search Screen - Advanced Search

Database - CINAHL Plus with Full Text 80,746

S31 (MH "Cardiovascular Diseases+") Expanders - Apply equivalent subjects

Search modes - Boolean/Phrase Interface - EBSCOhost Research Databases

Search Screen - Advanced Search

Database - CINAHL Plus with Full Text 680,677

S30 S22 OR S23 OR S24 OR S25 OR S26 OR S27 OR S28 OR S29 Expanders - Apply equivalent subjects

Search modes - Boolean/Phrase Interface - EBSCOhost Research Databases

Search Screen - Advanced Search

Database - CINAHL Plus with Full Text 98,525

S29 TI (("assisted living" or home or homes or house or household* or houses or community or facilit* or off-site or off-sites or "population-base*") and (setting* or location*)) Expanders - Apply equivalent subjects

Search modes - Boolean/Phrase Interface - EBSCOhost Research Databases

Search Screen - Advanced Search

Database - CINAHL Plus with Full Text 5,089

S28 TI (place or "health centre*" or "health center*" or incarcerat* or neighborhood* or neighbourhood* or "nursing home*" or penitentiar* or prison* or "rehabilitation center*" or "rehabilitation centre*" or "treatment center*" or "treatment centre*") Expanders - Apply equivalent subjects

Search modes - Boolean/Phrase Interface - EBSCOhost Research Databases

Search Screen - Advanced Search

Database - CINAHL Plus with Full Text 49,532

S27 (MM "Residential Facilities") Expanders - Apply equivalent subjects

Search modes - Boolean/Phrase Interface - EBSCOhost Research Databases

Search Screen - Advanced Search

Database - CINAHL Plus with Full Text 3,031

S26 (MM "Rehabilitation Centers") Expanders - Apply equivalent subjects

Search modes - Boolean/Phrase Interface - EBSCOhost Research Databases

Search Screen - Advanced Search

Database - CINAHL Plus with Full Text 3,039

S25 (MM "Population Health") Expanders - Apply equivalent subjects

Search modes - Boolean/Phrase Interface - EBSCOhost Research Databases

Search Screen - Advanced Search

Database - CINAHL Plus with Full Text 1,414

S24 (MM "Public Health") Expanders - Apply equivalent subjects

Search modes - Boolean/Phrase Interface - EBSCOhost Research Databases

Search Screen - Advanced Search

Database - CINAHL Plus with Full Text 33,571

S23 (MM "Correctional Facilities") Expanders - Apply equivalent subjects

Search modes - Boolean/Phrase Interface - EBSCOhost Research Databases

Search Screen - Advanced Search

Database - CINAHL Plus with Full Text 4,246

S22 (MM "Community Health Centers+") Expanders - Apply equivalent subjects

Search modes - Boolean/Phrase Interface - EBSCOhost Research Databases

Search Screen - Advanced Search

Database - CINAHL Plus with Full Text 3,129

S21 S7 OR S8 OR S9 OR S10 OR S11 OR S12 OR S13 OR S14 OR S15 OR S16 OR S17 OR S18 OR S19 OR S20 Expanders - Apply equivalent subjects

Search modes - Boolean/Phrase Interface - EBSCOhost Research Databases

Search Screen - Advanced Search

Database - CINAHL Plus with Full Text 874,778

S20 TI ( biomarker* or antibod* or antigen* or apolipoprotein* or "biological marker*" or "DNA methylation" or "dna sequenc*" or enzyme* or epigenetic* or "gene expression*" or "genetic marker*" or hormon* or mediator* or mRNA* or peptide* or protein* ) OR AB ( biomarker* or antibod* or antigen* or apolipoprotein* or "biological marker*" or "DNA methylation" or "dna sequenc*" or enzyme* or epigenetic* or "gene expression*" or "genetic marker*" or hormon* or mediator* or mRNA* or peptide* or protein* ) Expanders - Apply equivalent subjects

Search modes - Boolean/Phrase Interface - EBSCOhost Research Databases

Search Screen - Advanced Search

Database - CINAHL Plus with Full Text 496,210

S19 TI lipid* or marker* Expanders - Apply equivalent subjects

Search modes - Boolean/Phrase Interface - EBSCOhost Research Databases

Search Screen - Advanced Search

Database - CINAHL Plus with Full Text 196,476

S18 (MM "Lipids") Expanders - Apply equivalent subjects

Search modes - Boolean/Phrase Interface - EBSCOhost Research Databases

Search Screen - Advanced Search

Database - CINAHL Plus with Full Text 8,807

S17 (MM "RNA, Messenger") Expanders - Apply equivalent subjects

Search modes - Boolean/Phrase Interface - EBSCOhost Research Databases

Search Screen - Advanced Search

Database - CINAHL Plus with Full Text 1,040

S16 (MM "Epigenomics") Expanders - Apply equivalent subjects

Search modes - Boolean/Phrase Interface - EBSCOhost Research Databases

Search Screen - Advanced Search

Database - CINAHL Plus with Full Text 1,307

S15 (MM "Genomics") Expanders - Apply equivalent subjects

Search modes - Boolean/Phrase Interface - EBSCOhost Research Databases

Search Screen - Advanced Search

Database - CINAHL Plus with Full Text 4,890

S14 (MM "Antibodies+") Expanders - Apply equivalent subjects

Search modes - Boolean/Phrase Interface - EBSCOhost Research Databases

Search Screen - Advanced Search

Database - CINAHL Plus with Full Text 49,818

S13 (MM "Antigens+") Expanders - Apply equivalent subjects

Search modes - Boolean/Phrase Interface - EBSCOhost Research Databases

Search Screen - Advanced Search

Database - CINAHL Plus with Full Text 25,435

S12 (MM "Hormones+") Expanders - Apply equivalent subjects

Search modes - Boolean/Phrase Interface - EBSCOhost Research Databases

Search Screen - Advanced Search

Database - CINAHL Plus with Full Text 84,653

S11 (MM "Proteins+") Expanders - Apply equivalent subjects

Search modes - Boolean/Phrase Interface - EBSCOhost Research Databases

Search Screen - Advanced Search

Database - CINAHL Plus with Full Text 276,926

S10 (MM "Peptides+") Expanders - Apply equivalent subjects

Search modes - Boolean/Phrase Interface - EBSCOhost Research Databases

Search Screen - Advanced Search

Database - CINAHL Plus with Full Text 112,868

S9 (MM "Enzymes+") Expanders - Apply equivalent subjects

Search modes - Boolean/Phrase Interface - EBSCOhost Research Databases

Search Screen - Advanced Search

Database - CINAHL Plus with Full Text 73,451

S8 (MH "Biological Markers+") Expanders - Apply equivalent subjects

Search modes - Boolean/Phrase Interface - EBSCOhost Research Databases

Search Screen - Advanced Search

Database - CINAHL Plus with Full Text 94,507

S7 (MH "Blood Proteins+") Expanders - Apply equivalent subjects

Search modes - Boolean/Phrase Interface - EBSCOhost Research Databases

Search Screen - Advanced Search

Database - CINAHL Plus with Full Text 172,844

S6 S1 OR S2 OR S3 OR S4 OR S5 Expanders - Apply equivalent subjects

Search modes - Boolean/Phrase Interface - EBSCOhost Research Databases

Search Screen - Advanced Search

Database - CINAHL Plus with Full Text 3,440

S5 TI ( "blood blot*" or "blood spot*" or bloodspot* or "finger-stick blood") N3 (collect* or sampl* ) OR AB ( "blood blot*" or "blood spot*" or bloodspot* or "finger-stick blood") N3 (collect* or sampl* ) Expanders - Apply equivalent subjects

Search modes - Boolean/Phrase Interface - EBSCOhost Research Databases

Search Screen - Advanced Search

Database - CINAHL Plus with Full Text 457

S4 TI (("blood blot*" or "blood spot*" or bloodspot*) and ("filter paper*") ) OR AB (("blood blot*" or "blood spot*" or bloodspot*) and ("filter paper*") ) Expanders - Apply equivalent subjects

Search modes - Boolean/Phrase Interface - EBSCOhost Research Databases

Search Screen - Advanced Search

Database - CINAHL Plus with Full Text 80

S3 TI ( "Guthrie card*" or "Guthrie paper*" or "dried blood" or "dried whole blood spot*" or "dried whole bloodspot*" ) OR AB ( "Guthrie card*" or "Guthrie paper*" or "dried blood" or "dried whole blood spot*" or "dried whole bloodspot*" ) Expanders - Apply equivalent subjects

Search modes - Boolean/Phrase Interface - EBSCOhost Research Databases

Search Screen - Advanced Search

Database - CINAHL Plus with Full Text 1,126

S2 TI dbs Expanders - Apply equivalent subjects

Search modes - Boolean/Phrase Interface - EBSCOhost Research Databases

Search Screen - Advanced Search

Database - CINAHL Plus with Full Text 343

S1 (MM "Hematologic Tests") Expanders - Apply equivalent subjects

Search modes - Boolean/Phrase Interface - EBSCOhost Research Databases

Search Screen - Advanced Search

Database - CINAHL Plus with Full Text 1,856

# PsycInfo ( EBSCO)

Wednesday, April 24, 2024 8:09:33 PM

# Query Limiters/Expanders Last Run Via Results

S39 S37 OR S38 Expanders - Apply equivalent subjects

Search modes - Boolean/Phrase Interface - EBSCOhost Research Databases

Search Screen - Advanced Search

Database - APA PsycInfo 503

S38 (S20 AND S29 AND S36) Expanders - Apply equivalent subjects

Search modes - Boolean/Phrase Interface - EBSCOhost Research Databases

Search Screen - Advanced Search

Database - APA PsycInfo 472

S37 S5 AND S20 AND S36 Expanders - Apply equivalent subjects

Search modes - Boolean/Phrase Interface - EBSCOhost Research Databases

Search Screen - Advanced Search

Database - APA PsycInfo 31

S36 S30 OR S31 OR S32 OR S33 OR S34 OR S35 Expanders - Apply equivalent subjects

Search modes - Boolean/Phrase Interface - EBSCOhost Research Databases

Search Screen - Advanced Search

Database - APA PsycInfo 100,203

S35 TI ( "acute coronary syndrome" or "angina pectoris" or asystole* or atherosclero* or atherosclerotic or "cardiac arrest*" or "cardiac infarct*" or "cardiomyopath*" or "cardiovascular abnormal*" or "cardiovascular event*" or "cardiovascular infection*" or "cardiovascular outcome*" or "cerebral vascular accident*" or "cerebrovascular accident*" or "high cholesterol " or "coronary artery disease*" or "coronary artery obstruction*" or "coronary artery thrombos*" or "coronary occlusion" or endocarditis or "heart aneurysm*" or "heart arrest*" or "heart attack*" or "heart failure" or "heart infarct*" or "high blood pressure" or hypertension or hyperlipidemia or "low grade inflammation" or dyslipidemia or "myocardial failure*" or "myocardial infarct*" or "myocardial ischemia" or "myocardium failure " or "myocardium infarct*" or "percutaneous coronary intervention*" or pericarditis or "peripheral arterial disease" or stenocardia* or stroke* or "subclinical cardiovascular" or "vascular malformation*" or "venous insufficien*" or "ventricular dysfunction*" or "ventricular outflow obstruction*" ) OR AB ( "acute coronary syndrome" or "angina pectoris" or asystole* or atherosclero* or atherosclerotic or "cardiac arrest*" or "cardiac infarct*" or "cardiomyopath*" or "cardiovascular abnormal*" or "cardiovascular event*" or "cardiovascular infection*" or "cardiovascular outcome*" or "cerebral vascular accident*" or "cerebrovascular accident*" or "high cholesterol " or "coronary artery disease*" or "coronary artery obstruction*" or "coronary artery thrombos*" or "coronary occlusion" or endocarditis or "heart aneurysm*" or "heart arrest*" or "heart attack*" or "heart failure" or "heart infarct*" or "high blood pressure" or hypertension or hyperlipidemia or "low grade inflammation" or dyslipidemia or "myocardial failure*" or "myocardial infarct*" or "myocardial ischemia" or "myocardium failure " or "myocardium infarct*" or "percutaneous coronary intervention*" or pericarditis or "peripheral arterial disease" or stenocardia* or stroke* or "subclinical cardiovascular" or "vascular malformation*" or "venous insufficien*" or "ventricular dysfunction*" or "ventricular outflow obstruction*" ) Expanders - Apply equivalent subjects

Search modes - Boolean/Phrase Interface - EBSCOhost Research Databases

Search Screen - Advanced Search

Database - APA PsycInfo 68,508

S34 TI inflammation* Expanders - Apply equivalent subjects

Search modes - Boolean/Phrase Interface - EBSCOhost Research Databases

Search Screen - Advanced Search

Database - APA PsycInfo 4,292

S33 TI ( inflammat* n2 (heart or cardiac) ) OR AB ( inflammat* n2 (heart or cardiac) ) Expanders - Apply equivalent subjects

Search modes - Boolean/Phrase Interface - EBSCOhost Research Databases

Search Screen - Advanced Search

Database - APA PsycInfo 100

S32 MM "Inflammation" OR MM "Meningoradiculitis" OR MM "Neuroinflammation" Expanders - Apply equivalent subjects

Search modes - Boolean/Phrase Interface - EBSCOhost Research Databases

Search Screen - Advanced Search

Database - APA PsycInfo 13,127

S31 MM "Cerebrovascular Accidents" Expanders - Apply equivalent subjects

Search modes - Boolean/Phrase Interface - EBSCOhost Research Databases

Search Screen - Advanced Search

Database - APA PsycInfo 21,197

S30 DE "Cardiovascular Disorders" OR DE "Aneurysms" OR DE "Arteriosclerosis" OR DE "Blood Pressure Disorders" OR DE "Cerebrovascular Disorders" OR DE "Embolisms" OR DE "Heart Disorders" OR DE "Hemorrhage" OR DE "Hypertension" OR DE "Ischemia" OR DE "Thromboses" Expanders - Apply equivalent subjects

Search modes - Boolean/Phrase Interface - EBSCOhost Research Databases

Search Screen - Advanced Search

Database - APA PsycInfo 39,320

S29 S21 OR S22 OR S23 OR S24 OR S25 OR S26 OR S27 OR S28 Expanders - Apply equivalent subjects

Search modes - Boolean/Phrase Interface - EBSCOhost Research Databases

Search Screen - Advanced Search

Database - APA PsycInfo 335,732

S28 TI ( "assisted living" or home or homes or house or household* or houses or community or facilit* or off-site or off-sites or "population-base*") and (setting* or location* ) OR AB ( "assisted living" or home or homes or house or household* or houses or community or facilit* or off-site or off-sites or "population-base*") and (setting* or location* ) Expanders - Apply equivalent subjects

Search modes - Boolean/Phrase Interface - EBSCOhost Research Databases

Search Screen - Advanced Search

Database - APA PsycInfo 89,143

S27 TI ( place or "health centre*" or "health center*" or incarcerat* or neighborhood* or neighbourhood* or "nursing home*" or penitentiar* or prison* or "rehabilitation center*" or "rehabilitation centre*" or "treatment center*" or "treatment centre*" ) OR AB ( place or "health centre*" or "health center*" or incarcerat* or neighborhood* or neighbourhood* or "nursing home*" or penitentiar* or prison* or "rehabilitation center*" or "rehabilitation centre*" or "treatment center*" or "treatment centre*" ) Expanders - Apply equivalent subjects

Search modes - Boolean/Phrase Interface - EBSCOhost Research Databases

Search Screen - Advanced Search

Database - APA PsycInfo 220,770

S26 MM "Residential Care Institutions" OR MM "Halfway Houses" OR MM "Hospitals" OR MM "Nursing Homes" OR MM "Orphanages" Expanders - Apply equivalent subjects

Search modes - Boolean/Phrase Interface - EBSCOhost Research Databases

Search Screen - Advanced Search

Database - APA PsycInfo 25,998

S25 MM "Rehabilitation Centers" OR MM "Sheltered Workshops" Expanders - Apply equivalent subjects

Search modes - Boolean/Phrase Interface - EBSCOhost Research Databases

Search Screen - Advanced Search

Database - APA PsycInfo 604

S24 MM "Population Health" Expanders - Apply equivalent subjects

Search modes - Boolean/Phrase Interface - EBSCOhost Research Databases

Search Screen - Advanced Search

Database - APA PsycInfo 577

S23 MM "Public Health" OR MM "Community Health" OR MM "Community Mitigation" OR MM "Public Health Attitudes" OR MM "Public Health Campaigns" OR MM "Public Health Research" OR MM "Public Mental Health" Expanders - Apply equivalent subjects

Search modes - Boolean/Phrase Interface - EBSCOhost Research Databases

Search Screen - Advanced Search

Database - APA PsycInfo 24,536

S22 MM "Correctional Institutions" OR MM "Prisons" OR MM "Reformatories" Expanders - Apply equivalent subjects

Search modes - Boolean/Phrase Interface - EBSCOhost Research Databases

Search Screen - Advanced Search

Database - APA PsycInfo 8,553

S21 MM "Community Mental Health Centers" Expanders - Apply equivalent subjects

Search modes - Boolean/Phrase Interface - EBSCOhost Research Databases

Search Screen - Advanced Search

Database - APA PsycInfo 1,394

S20 S6 OR S7 OR S8 OR S9 OR S10 OR S11 OR S12 OR S13 OR S14 OR S15 OR S16 OR S17 OR S18 OR S19 Expanders - Apply equivalent subjects

Search modes - Boolean/Phrase Interface - EBSCOhost Research Databases

Search Screen - Advanced Search

Database - APA PsycInfo 268,308

S19 TI ( biomarker* or antibod* or antigen* or apolipoprotein* or "biological marker*" or "DNA methylation" or "dna sequenc*" or enzyme* or epigenetic* or "gene expression*" or "genetic marker*" or hormon* or mediator* or mRNA* or peptide* or protein*) ) OR AB ( biomarker* or antibod* or antigen* or apolipoprotein* or "biological marker*" or "DNA methylation" or "dna sequenc*" or enzyme* or epigenetic* or "gene expression*" or "genetic marker*" or hormon* or mediator* or mRNA* or peptide* or protein*) ) Expanders - Apply equivalent subjects

Search modes - Boolean/Phrase Interface - EBSCOhost Research Databases

Search Screen - Advanced Search

Database - APA PsycInfo 229,770

S18 TI (lipid* or marker*) Expanders - Apply equivalent subjects

Search modes - Boolean/Phrase Interface - EBSCOhost Research Databases

Search Screen - Advanced Search

Database - APA PsycInfo 9,120

S17 MM "Lipids" OR MM "Fatty Acids" OR MM "Gangliosides" OR MM "Lipopolysaccharide" OR MM "Lipoproteins" Expanders - Apply equivalent subjects

Search modes - Boolean/Phrase Interface - EBSCOhost Research Databases

Search Screen - Advanced Search

Database - APA PsycInfo 6,615

S16 MM "mRNA" Expanders - Apply equivalent subjects

Search modes - Boolean/Phrase Interface - EBSCOhost Research Databases

Search Screen - Advanced Search

Database - APA PsycInfo 1,991

S15 MM "Epigenetics" Expanders - Apply equivalent subjects

Search modes - Boolean/Phrase Interface - EBSCOhost Research Databases

Search Screen - Advanced Search

Database - APA PsycInfo 2,834

S14 MM "Genomics" OR MM "Genome" OR MM "Proteomics" Expanders - Apply equivalent subjects

Search modes - Boolean/Phrase Interface - EBSCOhost Research Databases

Search Screen - Advanced Search

Database - APA PsycInfo 5,565

S13 MM "Antibodies" OR MM "Monoclonal Antibodies" Expanders - Apply equivalent subjects

Search modes - Boolean/Phrase Interface - EBSCOhost Research Databases

Search Screen - Advanced Search

Database - APA PsycInfo 4,367

S12 MM "Antigens" Expanders - Apply equivalent subjects

Search modes - Boolean/Phrase Interface - EBSCOhost Research Databases

Search Screen - Advanced Search

Database - APA PsycInfo 925

S11 MM "Hormones" OR MM "Adrenal Cortex Hormones" OR MM "Adrenal Medulla Hormones" OR MM "Cholecystokinin" OR MM "Corticotropin Releasing Factor" OR MM "Epinephrine" OR MM "Ghrelin" OR MM "Glucagon" OR MM "Gonadotropic Hormones" OR MM "Insulin" OR MM "Leptin" OR MM "Melatonin" OR MM "Orexin" OR MM "Parathyroid Hormone" OR MM "Pituitary Hormones" OR MM "Progestational Hormones" OR MM "Sex Hormones" OR MM "Thyroid Hormones" Expanders - Apply equivalent subjects

Search modes - Boolean/Phrase Interface - EBSCOhost Research Databases

Search Screen - Advanced Search

Database - APA PsycInfo 20,425

S10 MM "Proteins" OR MM "Apolipoprotein E" OR MM "Apolipoproteins" OR MM "Beta Amyloid" OR MM "Blood Proteins" OR MM "Cell Adhesion Molecules" OR MM "CLOCK Gene" OR MM "Endorphins" OR MM "Globulins" OR MM "Growth Factor" OR MM "Interferons" OR MM "Ion Channel" OR MM "Neurofibrillary Tangles" OR MM "Neurotransmitter Transporters" OR MM "Prion" OR MM "Rhodopsin" OR MM "Synaptotagmin" OR MM "Tau Proteins" Expanders - Apply equivalent subjects

Search modes - Boolean/Phrase Interface - EBSCOhost Research Databases

Search Screen - Advanced Search

Database - APA PsycInfo 40,745

S9 MM "Peptides" OR MM "Angiotensin" OR MM "Bombesin" OR MM "Cholecystokinin" OR MM "Corticotropin Releasing Factor" OR MM "Endogenous Opiates" OR MM "Ghrelin" OR MM "Insulin-like Growth Factor" OR MM "Leptin" OR MM "Melanocyte Stimulating Hormone" OR MM "Nerve Growth Factor" OR MM "Neuropeptides" OR MM "Neurotensin" OR MM "Somatostatin" OR MM "Tachykinins" Expanders - Apply equivalent subjects

Search modes - Boolean/Phrase Interface - EBSCOhost Research Databases

Search Screen - Advanced Search

Database - APA PsycInfo 15,145

S8 MM "Enzymes" OR MM "Adenylyl Cyclase" OR MM "Decarboxylases" OR MM "Dehydrogenases" OR MM "Esterases" OR MM "Hydroxylases" OR MM "Isozymes" OR MM "Kinases" OR MM "Oxidases" OR MM "Phosphatases" OR MM "Phosphodiesterase" OR MM "Phosphorylases" OR MM "Proteinases" OR MM "Transferases" Expanders - Apply equivalent subjects

Search modes - Boolean/Phrase Interface - EBSCOhost Research Databases

Search Screen - Advanced Search

Database - APA PsycInfo Display

S7 MM "Biological Markers" Expanders - Apply equivalent subjects

Search modes - Boolean/Phrase Interface - EBSCOhost Research Databases

Search Screen - Advanced Search

Database - APA PsycInfo Display

S6 DE "Blood Proteins" OR DE "Hemoglobin" OR DE "Immunoglobulins" OR DE "Serum Albumin" Expanders - Apply equivalent subjects

Search modes - Boolean/Phrase Interface - EBSCOhost Research Databases

Search Screen - Advanced Search

Database - APA PsycInfo Display

S5 S1 OR S2 OR S3 OR S4 Expanders - Apply equivalent subjects

Search modes - Boolean/Phrase Interface - EBSCOhost Research Databases

Search Screen - Advanced Search

Database - APA PsycInfo 664

S4 TI ( "blood blot*" or "blood spot*" or bloodspot* or "finger-stick blood") n3 (collect* or sampl* ) OR AB ( "blood blot*" or "blood spot*" or bloodspot* or "finger-stick blood") n3 (collect* or sampl* ) Expanders - Apply equivalent subjects

Search modes - Boolean/Phrase Interface - EBSCOhost Research Databases

Search Screen - Advanced Search

Database - APA PsycInfo Display

S3 TI ( "blood blot*" or "blood spot*" or bloodspot*) and "filter paper*" ) OR AB ( "blood blot*" or "blood spot*" or bloodspot*) and "filter paper*" ) Expanders - Apply equivalent subjects

Search modes - Boolean/Phrase Interface - EBSCOhost Research Databases

Search Screen - Advanced Search

Database - APA PsycInfo Display

S2 TI ( "Guthrie card*" or "Guthrie paper*" or "dried blood" or "dried whole blood spot*" or "dried whole bloodspot*" ) OR AB ( "Guthrie card*" or "Guthrie paper*" or "dried blood" or "dried whole blood spot*" or "dried whole bloodspot*" ) Expanders - Apply equivalent subjects

Search modes - Boolean/Phrase Interface - EBSCOhost Research Databases

Search Screen - Advanced Search

Database - APA PsycInfo Display

S1 TI dbs Expanders - Apply equivalent subjects

Search modes - Boolean/Phrase Interface - EBSCOhost Research Databases

Search Screen - Advanced Search

Database - APA PsycInfo Display
